# Relationship between experiencing a challenge or delay accessing contraception and contraceptive self-efficacy: Data from a 2022 nationally representative online survey

**DOI:** 10.1186/s12978-025-02003-3

**Published:** 2025-04-15

**Authors:** Alex Schulte, Ariana H. Bennett, Jennet Arcara, Jamie Bardwell, Aisha Chaudhri, Laura Davis, Brittni Frederiksen, Elizabeth Jones, Catherine Labiran, Raegan McDonald-Mosley, Whitney Rice, Tara Stein, Ena Suseth Valladares, Kari White, Cassondra Marshall, Anu Manchikanti Gomez

**Affiliations:** 1 Sexual Health and Reproductive Equity Program, School of Social Welfare, University of California, 120 Haviland Hall MC 7400, Berkeley, CA 94720-7400 USA; 2 Santa Clara University, Santa Clara, CA USA; 3 Converge, Jackson, MS USA; 4 Illinois Caucus for Adolescent Health, Chicago, IL USA; 5 Advocates for Youth, Washington, DC USA; 6 Kaiser Family Foundation, San Francisco, CA USA; 7 National Family Planning & Reproductive Health Association, Washington, DC USA; 8 Independent Consultant, Brooklyn, NY USA; 9 Power to Decide, Washington, DC USA; 10 Center for Reproductive Health Research in the Southeast (RISE), Emory University, Atlanta, GA USA; 11 NYC Department of Health and Mental Hygiene, New York, NY USA; 12 Nursing Student, Los Angeles, CA USA; 13 Resound Research for Reproductive Health, Austin, TX USA; 14 School of Public Health, University of California, Berkeley, CA USA

**Keywords:** Contraception, Self-efficacy, Access barriers, Person-centered care

## Abstract

**Background:**

Previous research has found self-efficacy is associated with reproductive health behaviors and outcomes. However, few studies have quantitatively examined the relationship between barriers accessing contraception and self-efficacy. In addition, existing population-level metrics of contraceptive access tend to focus on method availability, uptake, and use, rather than people’s self-defined needs and preferences. This study uses person-centered metrics to assess the relationship between experiencing a recent challenge or delay obtaining desired contraception and two measures of contraceptive self-efficacy.

**Methods:**

In 2022, we fielded a nationally representative survey in English and Spanish using NORC’s AmeriSpeak panel, surveying non-sterile 15- to 44-year-olds assigned female sex at birth in the U.S. We describe common challenges and delays obtaining contraception and present the distribution of experiencing a challenge or delay obtaining contraception by key sociodemographic and reproductive health characteristics. We also conduct logistic regression analyses to investigate associations between experiencing a challenge/delay and two measures of contraceptive self-efficacy: confidence in obtaining a desired method when wanted and perceived ease of switching methods when wanted.

**Results:**

Among the analytic sample of respondents who had ever used or tried to obtain a contraceptive product, device, or procedure (unweighted n = 2,771), about 14% experienced a challenge/delay obtaining desired contraception in the past year. The most common reasons for challenges or delays were related to logistics (transportation, childcare, scheduling; 38.2%) and cost/insurance coverage (35.8%). Among those who experienced a challenge/delay obtaining desired contraception (unweighted n = 301), higher proportions were younger, identified as non-heterosexual, had lower educational attainment, and could afford smaller emergency expenses compared to the subgroup that did not experience a challenge/delay. Experiencing a challenge/delay was associated with decreased odds of feeling very or somewhat confident in obtaining a desired method (aOR 0.14; 95% CI 0.07, 0.25) and decreased odds of feeling it would be very or somewhat easy to switch contraceptive methods (aOR 0.48; 95% CI 0.33, 0.71).

**Conclusions:**

Eliminating barriers to contraceptive care is crucial to achieving person-centered access. Our research suggests that experiencing a challenge/delay has implications not only for recent contraceptive access but also influences self-efficacy, potentially inhibiting future ability to obtain and use desired contraception.

## Background

Person-centered contraceptive access is necessary to achieve sexual and reproductive health equity—defined as all persons, across the range of age, gender, race, and other intersecting identities, having what they need to access and attain their highest level of sexual and reproductive health (SRH) [1–4]. However, current approaches to measuring contraceptive access focus on use and services provided and do not utilize a person-centered framework, in which an individual’s experiences, values, and preferences are prioritized and guide decision-making [3, 5–9]. Understanding individuals' perspectives on the challenges to accessing contraception is an important step in alleviating those challenges and promoting person-centered care. Despite advances in method availability and insurance coverage, individuals still report difficulty obtaining contraception. A 2023 study in three states found between 10 and 19% of contraceptive users reported a delay or trouble obtaining contraception in the past 12 months [10]. Previous research suggests that financial barriers are most common [10–16]. Other frequent barriers include those related to logistics (e.g., transportation and scheduling), lack of availability at a clinic or facility, provider bias, insufficient information, and privacy concerns [10, 16–19]. Contraceptive access barriers disproportionately affect groups already experiencing systemic inequities based on age, race, sexual orientation, and income [11, 13, 18, 20–24].

Accessing contraception is a multi-step process. Often, this process includes frequent engagement with the healthcare system, which can exacerbate and compound barriers. Actions necessary to access contraception typically include identifying one’s contraceptive needs (ideally using relevant and scientifically accurate information), selecting preferred method(s), seeking care from a healthcare provider (either in-person or via telehealth), obtaining and using desired method(s), and switching or discontinuing methods as desired. Many contraceptive methods still require regular engagement with healthcare professionals for continued use. For example, the oral contraceptive pill (one of the most commonly used methods in the U.S. [25, 26]) is generally taken daily, so an adequate supply is necessary for consistent and effective use. However, studies estimate about one-third of short-acting hormonal contraception users (e.g., pill, patch, ring or shot) have missed using their birth control because they were unable to obtain their next supply in time [16, 27]. A 2022 survey found 32% of contraceptive pill users received 1 or 2 (monthly) packs at a time, and only 6% received 6 or more packs at a time [16]. This occurs despite clinical guidelines that recommend prescribing a full year of contraception [28, 29] and many state policies that require public and/or commercial insurers to cover an extended supply (usually 12 months) of contraception [28].

Switching contraceptive methods is a common and inherent part of the SRH journey [30–32]. Recent studies have estimated about a quarter of contraceptive users would like to be using a different form of birth control [16, 24], and a 2023 study of contraceptive users who were patients at community health centers found over 20% switched methods over the 4-year observation period [32]. However, evidence suggests that individuals face several obstacles—in some cases, including provider bias—when attempting to switch or discontinue methods, especially when they desire to discontinue long-acting reversible contraception (LARC) [32–35].

Self-efficacy, defined as confidence in executing actions or behaviors for desired outcomes, is needed to overcome barriers inherent to the U.S. healthcare system and maintain consistent contraceptive access [36, 37]. Theoretical frameworks such as Levesque et al.’s model for patient-centered healthcare access [3] and Bandura’s social cognitive theory [38, 39] highlight that self-efficacy plays a key role in enabling individuals to take actions necessary to access healthcare. Previous research has found self-efficacy is associated with general health [40–43] and specific SRH-related [36, 37, 44–48] behaviors and outcomes. Additionally, social and structural environments strongly influence self-efficacy; researchers have found that individuals who identify as Black, Indigenous, and people of color (BIPOC), have low-incomes, and have lower educational attainment generally score lower on self-efficacy measures [36, 49–51]. Evidence also suggests that higher self-efficacy is associated with increased use of contraception [36, 37, 45, 52]. However, few studies have quantitatively examined the association between barriers accessing contraception and self-efficacy. In addition, existing population-level metrics of contraceptive access tend to focus on method availability, uptake, and use, rather than people’s self-defined needs and preferences [53, 54].

This study assesses the relationship between experiencing a recent challenge or delay obtaining desired contraception and contraceptive self-efficacy. We also examine sociodemographic and reproductive health characteristics associated with experiencing a challenge or delay. This study aims to contribute data on the types and frequency of barriers to timely access to desired contraception and novel insights regarding the impact on self-efficacy. These insights can inform strategies to overcome contraceptive barriers, which is especially important in the current U.S. policy environment, given the loss of a constitutional right to abortion care and increasing threats to SRH equity [55, 56].

## Methods

This study is part of the Person-Centered Contraceptive Access Metrics project [57], a stakeholder-engaged project that aimed to develop person-centered measures of contraceptive access to inform policy, research, and funding directions, while also disrupting norms in knowledge production that prioritize the perspective of academic researchers. Additional details about the project have been previously published [24, 27, 57]. We used a multi-step process to develop, test, and deploy this study's survey instrument. First, we developed survey questions to quantify key aspects of person-centered contraceptive access using insights from a literature review and interviews with stakeholders from diverse sectors (e.g., reproductive justice, advocacy, clinical care, public health). We then sought expert review on the survey draft and conducted 33 cognitive interviews in English and Spanish to understand how survey respondents would make sense of the questions and whether they captured the intended constructs. Next, we fielded these refined questions in a nationally representative survey in partnership with NORC at the University of Chicago. Finally, we convened a Working Group to develop and select priority metrics. One of the four key metrics prioritized by the Working Group was experiencing a challenge or delay obtaining desired contraception in the past year. The Committee for the Protection of Human Subjects at the University of California, Berkeley and the Institutional Review Board at NORC at the University of Chicago approved the study protocol.

### Data source

We fielded a nationally representative online survey between January and March 2022 using NORC’s AmeriSpeak Panel. AmeriSpeak is a multistage probability sample constructed to represent the U.S. household population—including over 54,000 members and covering an estimated 97% of U.S. households [58]. Before survey distribution, we completed a power analysis to determine the target sample size with sufficient statistical power so that descriptive analyses could detect statistically significant differences between subgroups. Panelists were eligible for the survey if they were: ages 15–44, assigned female sex at birth, not known to be sterile, and able to complete the self-administered survey in English or Spanish. All panelists aged 18 and older who met the age and sex criteria were invited to provide informed consent and complete an initial screening. The parents of panelists aged 15–17 first provided informed consent before their children provided their own informed assent and completed screening. Eligible participants continued to the full survey, which took approximately 25 min to complete. Approximately 97% of panelists who completed the initial screening and met eligibility criteria also completed the full survey. Respondents received the equivalent of $8 in “AmeriSpeak points” upon survey completion.

### Sample

The analytic sample includes respondents who had ever used or tried to obtain a contraceptive method that is a product, device, or procedure (unweighted n = 2,798). We defined contraceptive products, devices, and procedures as the following: oral contraceptive pill, patch, ring, shot, implant, hormonal and copper intrauterine devices (IUDs), emergency contraception, vasectomy, condoms, gel (e.g., Phexxi), spermicide, diaphragm, cervical cap, and sponge. Vasectomy refers to a partner’s vasectomy. We excluded respondents who skipped 3 or more key survey questions used in this analysis. Statistical tests indicated these responses were missing completely at random. NORC constructed survey weights to account for differences between the U.S. population and the survey sample based on age, education, race/ethnicity, marital status, number of children in household, and age by race/ethnicity. The final analytic sample (unweighted n = 2,771), when weighted, represents approximately 40.8 million 15- to 44-year-olds who met inclusion criteria.

### Measures

Our first measure of interest was experiencing a challenge or delay obtaining a desired contraceptive method in the past year. We categorized respondents as having experienced a challenge/delay if they answered yes to the following question: “In the past 12 months, have you encountered any challenges or delays in getting the birth control method you wanted?” (response options: yes, no). Respondents who answered “yes” were then shown a list of types of challenges (based on previous literature [10, 17, 18]) and asked to select all that apply.

Second, we operationalized contraceptive self-efficacy using two items: confidence in obtaining a desired method when wanted and perceived ease of switching methods when wanted. To ascertain confidence, we asked, “In general, how confident are you that you can get the birth control method that you want, when you want it?” (response options: very confident, somewhat confident, not confident). We created a dichotomous variable indicating if the respondent felt very or somewhat confident vs. not confident. To ascertain perceived ease in switching methods, we asked current contraceptive users, “Overall, how difficult or easy do you think it will be to switch to a different birth control method when you want to?” (response options: very easy, somewhat easy, somewhat difficult, very difficult, I’m not sure). We created a dichotomous variable indicating if the respondent felt it would be very or somewhat easy to switch methods vs. not. Taken together, these measures capture key components of contraceptive self-efficacy as it relates to person-centered access: confidence in defining and obtaining desired method(s) and switching when wanted [3, 36, 37].

Our survey also included several sociodemographic and reproductive health characteristics. Sociodemographic measures included age, race/ethnicity, survey language, sexual orientation, highest education completed, insurance type, urbanicity, and employment status. In addition, we measured largest affordable emergency expense using the question: “Based on your current financial situation, what is the largest emergency expense that you could pay right now using cash or money in your checking/savings account?” (response options: I could not pay for any emergency expense, $1 to $49, $50 to $99, $100 to $199, $200 to $299, $300 to $399, over $400). We believe this metric more accurately and directly measured a respondent’s financial situation compared to other variables such as household income due to variations in cost of living and household size.

For reproductive health characteristics, we defined current contraceptive use as any method used in the past month, and respondents could indicate use of multiple methods. We created a mutually exclusive categorical variable for current contraceptive method type; if respondents selected more than one method, the most effective method [59] was used to create this variable. Methods were grouped into the following six categories: (1) no method; (2) coital-based withdrawal or fertility awareness-based contraceptive methods (FABM); (3) coital-based product/device (condoms, spermicide, sponge, diaphragm, cervical cap, Phexxi, or emergency contraception); (4) short-acting reversible contraception (SARC), including the pill, patch, ring, and shot; (5) long-acting reversible contraception (LARC), including IUDs and implants; and (6) vasectomy. We also created a “contraceptive user status" variable to capture if respondents were content with their current contraceptive use or non-use. Current users were categorized as one of the following: using their preferred method (content current user); wanting to use a different method, no method, or stop using any of their methods as soon as possible; or uncertain about if they would like to use a different method. Respondents who were not using contraception at the time of the survey were categorized as one of the following: content non-user (does not want to use contraception); prospective user (wants to use a method of contraception); or uncertain about if they would like to use contraception. In addition, respondents who indicated they had ever discussed contraception with a healthcare provider were asked whether they had ever experienced nine types of discrimination due to race, ancestry, or national origin in family planning settings [60]. We created a categorical variable representing the number of discrimination types ever experienced (0, 1–4, 5–9). Last, we used the Person-Centered Contraceptive Counseling (PCCC) measure [61], in which respondents rated their most recent contraceptive care provider on four aspects of person-centered care. Following scoring conventions for the measure, we used a binary variable for receipt of PCCC, which denotes “excellent” ratings for all four items.

### Statistical analyses

We present descriptive statistics for sociodemographic and reproductive health characteristics of the analytic sample, as well as the distribution of experiencing a challenge/delay obtaining contraception by key sociodemographic and reproductive health characteristics. Bivariate tests were conducted using the Rao-Scott corrected chi-square test [62, 63]. In addition, we used logistic regression analyses to investigate associations between experiencing a challenge/delay obtaining desired contraception in the past year and measures of contraceptive self-efficacy. Adjusted models control for age, race/ethnicity, sexual orientation, educational attainment, insurance, employment status, urbanicity, and largest affordable emergency expense. We conducted all analyses using Stata (version 17.0) and applied *svy* commands to account for weighting and complex survey design. Statistical significance was determined using a threshold of p < 0.05. All reported proportions are weighted.

## Results

The sociodemographic and reproductive health characteristics of the analytic sample, those who had ever used or tried to obtain a contraceptive product, device, or procedure and met other inclusion criteria (unweighted n = 2,771), are presented in Tables 1 and 2, respectively. About half the sample was between the ages of 18–29 (48.9%), and slightly over half identified as white (53.8%; Table 1). A majority identified as straight/heterosexual (80.6%), and the highest education level completed was high school or less for about one-third of respondents (33.5%). As shown in Table 2, almost 1 in 6 (13.7%) experienced a challenge or delay obtaining desired contraception in the 12 months prior to survey administration. Nearly all were somewhat or very confident (94.8%) they could obtain contraception when wanted, and over half of current users (61.9%) thought it would be very or somewhat easy to switch contraceptive methods. Regarding contraceptive user status, 48.7% were content current users, meaning they were using a preferred contraceptive method. About half (51.9%) of respondents had previously experienced one or more types of discrimination in a family planning setting, and the majority (64.4%) did not receive PCCC from the most recent healthcare provider seen for contraception.

**Table 1:** Sociodemographic characteristics of a 2022 national sample of 15- to 44-year-olds who had ever used or tried to obtain contraception (unweighted n = 2,771)

Variable	Unweighted n	Weighted %
Age, years		
15–17	85	6.1
18–24	268	26.1
25–29	607	22.8
30–34	715	20.6
35–39	631	13.7
40–44	465	10.8
Race/ethnicity		
White only	1,553	53.8
Latinx/Hispanic	484	20.9
Black only	382	14.8
Asian/Pacific Islander only	176	6.5
Multiracial, not including Latinx/Hispanic	139	3.4
Another race/ethnicity only	37	0.7
Survey language		
English	2,719	98.0
Spanish	52	2.0
Sexual orientation		
Straight/heterosexual	2,344	80.6
Bisexual	289	13.9
Gay/lesbian	48	2.1
Queer	53	2.0
Something else	28	1.5
Missing	9	0.3
Highest education completed		
Less than high school	165	12.8
High school or equivalent	339	20.7
Vocational or technical school, some college, or associate’s degree	987	32.6
Bachelor's degree	829	22.9
Post graduate study or professional degree	451	11.0
Insurance type		
Commercial (e.g., employer-based, direct purchase, health insurance exchange)	1,911	63.5
State Medicaid or CHIP	480	21.0
Other public insurance (including Medicare, military/VA, IHS)	124	5.0
None	176	6.7
Don't know	71	3.8
Missing	9	0.2
Employment status		
Working full time	1,569	52.5
Working part time	458	17.8
Not working for pay	728	29.4
Other	9	0.4
Missing	7	0.3
Urbanicity		
Urban	1,156	38.9
Suburban	1,198	45.5
Rural	417	15.6
Largest affordable emergency expense		
I could not pay for any emergency expense	429	19.5
$1–99	310	13.1
$100–399	539	21.6
Over $400	1,472	45.9
Missing	21	0.6

*CHIP* Children’s Health Insurance Program, *VA* Veteran’s Administration, *IHS* Indian Health Service

**Table 2:** Reproductive health characteristics of a 2022 national sample of 15- to 44-year-olds who had ever used or tried to obtain contraception (unweighted n = 2,771)

	Unweighted n	Weighted %
Experienced a challenge/delay obtaining desired contraception in the past year		
Yes	301	13.7
No	2,486	86.3
Confidence in obtaining desired contraception when wanted		
Not confident	123	5.2
Somewhat confident	919	37.5
Very confident	1,714	57.3
Missing	15	0.5
Perceived ease of switching contraceptive methods when wanted		
Very difficult	155	7.2
Somewhat difficult	426	21.2
Somewhat easy	746	37.5
Very easy	546	24.4
I'm not sure	170	9.7
Not current contraceptive user	713	24.5
Missing	15	0.3
Current contraceptive method*		
No method	713	23.9
Coital-based FABM or withdrawal	339	12.9
Coital-based condoms, spermicide, sponge, diaphragm, cervical cap, Phexxi, EC	375	13.5
SARC (pill, patch, ring, shot)	618	26.5
LARC (IUD, implant)	505	18.4
Vasectomy	221	4.8
Contraceptive user status		
Current user	2053	75.9
Using preferred method (content current user)	1328	48.7
Wants to use a different method, no method, or stop ASAP	456	17.6
Uncertain current user	269	9.6
Current non-user	711	23.9
Content non-user (does not want to use a method)	439	14.5
Prospective user (wants to be using a method)	144	5.1
Uncertain non-user	128	4.3
Missing	7	0.2
Number of types of discrimination ever experienced in a family planning setting		
0 types	1,284	48.1
1–4 types	708	27.5
5–9 types	616	24.4
Did not report ever discussing contraception with a healthcare provider	100	4.4
Missing	63	2.2
Received PCCC from most recent healthcare provider seen for contraception		
Yes	1,042	35.6
No	1,617	64.4
Did not report ever discussing contraception with a healthcare provider	100	4.4
Missing	12	0.4

*EC* emergency contraception, *FABM* fertility awareness-based methods, *SARC* short-acting reversible contraception, *LARC* long-acting reversible contraception, *PCCC* Person-Centered Contraceptive Counseling, *ASAP* as soon a possible

^*^Some respondents were currently using more than one contraceptive method. Most effective method reported. Abstinence not considered a contraceptive method

Among respondents who indicated they had experienced one or more challenges or delays (unweighted n = 301), the most common types of challenges/delays were related to logistics (38.2%), including transportation, childcare, and/or scheduling, followed by finances (35.8%), including out-of-pocket cost and/or insurance coverage (Table 3). 21% experienced a challenge/delay because the method was not available at the facility due to supply constraints and/or religious affiliation. Almost 1 in 5 (19.2%) experienced a challenge/delay related to the provider or staff (poor treatment or unwillingness to provide a method).

**Table 3:** Type of challenge or delay experienced among respondents reporting a challenge or delay in the last 12 months (unweighted n = 301)

Type of challenge or delay	Unweighted n	Weighted %
Cost or insurance	113	35.8
Logistics (transportation, childcare, scheduling)	104	38.2
Facility didn't have a preferred method	55	21.0
Clinician unwilling to provide method or poor treatment by clinician/staff	58	19.2
Did not know where to get contraception	20	5.5
Privacy or confidentiality concerns	10	3.2
Something else	45	11.4

Categories are not mutually exclusive; some respondents experienced more than one type of challenge or delay

In Table 4, we present bivariate analyses showing the distribution of experiencing a challenge/delay obtaining desired contraception by key sociodemographic characteristics. There were differences in the distributions by age, sexual orientation, highest education completed, and largest affordable emergency expense. The youngest groups in our sample (15–17 and 18–24 year-olds) comprised nearly half (47.0%) of those who experienced a challenge/delay compared to 29.8% of those who did not (p < 0.001). Regarding sexual orientation, those who did not identify as heterosexual comprised a higher portion (28.9%) of those who experienced a challenge/delay compared to the subgroup that did not experience a challenge/delay (17.9%; p = 0.004). Among those who experienced a challenge/delay, 25.9% had a bachelor’s degree or higher, compared to 35.3% of those who did not experience a challenge/delay (p = 0.049). Last, 34.9% of those who experienced a challenge/delay could pay for an emergency expense over $400, compared 47.6% of those who did not experience a challenge/delay (p = 0.022).

**Table 4:** Bivariate analyses of sociodemographic characteristics and experiencing a challenge or delay obtaining desired contraception among a 2022 national sample of 15- to 44-year-olds who had ever used or tried to obtain contraception (unweighted n = 2,771)

Variable	Experienced a challenge or delay		
	Yes weighted %	No weighted %	p-value
Age, years			0.001
15–17	7.6	5.8	
18–24	39.4	23.9	
25–29	18.3	23.5	
30–34	18.7	20.9	
35–39	10.4	14.2	
40–44	5.7	11.6	
Race/ethnicity			0.196
White only	59.6	52.9	
Latinx/Hispanic	21.9	20.7	
Black only	11.0	15.4	
Asian/Pacific Islander only	4.7	6.8	
Multiracial, not including Latinx/Hispanic	2.2	3.6	
Another race/ethnicity only	0.6	0.7	
Survey language			0.8000
English	98.3	97.9	
Spanish	1.7	2.1	
Sexual orientation			0.004
Straight/heterosexual	71.1	82.1	
Bisexual	23.6	12.3	
Gay/lesbian	1.1	2.2	
Queer	2.1	2.0	
Something else	2.1	1.4	
Highest education completed			0.049
Less than high school	15.4	12.3	
High school or equivalent	17.7	21.2	
Vocational or technical school, some college, or associate’s degree	41.0	31.2	
Bachelor's degree	18.4	23.6	
Post graduate study or professional degree	7.5	11.6	
Insurance type			0.746
Commercial (e.g., employer-based, direct purchase, health insurance exchange)	67.1	62.9	
State Medicaid or CHIP	20.8	21.1	
Other public insurance (including Medicare, military/VA, IHS)	3.8	5.2	
None	5.6	6.9	
Don't know	2.7	4.0	
Employment status			0.309
Working full time	47.4	53.3	
Working part time	17.7	17.8	
Not working for pay	34.9	28.5	
Other	0.0	0.5	
Urbanicity			0.310
Urban	44.1	38.1	
Suburban	42.8	45.9	
Rural	13.1	16.0	
Largest affordable emergency expense			0.022
I could not pay for any emergency expense	26.3	18.4	
$1–99	14.6	12.8	
$100–399	24.3	21.2	
Over $400	34.9	47.6	

*CHIP* Children’s Health Insurance Program, *VA* Veteran’s Administration, *IHS* Indian Health Service

The distribution of experiencing a challenge/delay by key reproductive health characteristics is presented in Table 5. A higher portion (47.4%) of those who experienced a challenge/delay were current SARC users compared to those who did not experience a challenge/delay (23.1%; p < 0.001). Additionally, those using a preferred contraceptive method comprised a smaller portion (34.1%) of those who experienced a challenge/delay compared to the subgroup who did not experience a challenge/delay (51.2%; p < 0.001). Of those who experienced a challenge/delay, 72.0% had also experienced 1 or more types of discrimination in a family planning setting; of those who did not experience a challenge or delay, 48.5% experienced 1 or more types of discrimination (p < 0.001). Lastly, those who did not receive PCCC from the most recent healthcare provider they saw for contraception comprised a larger portion (75.6%) of those who experienced a challenge/delay compared to those who did not experience a challenge/delay (62.5%; p = 0.002).

**Table 5:** Bivariate analyses of reproductive health characteristics and experiencing a challenge or delay obtaining desired contraception among a 2022 national sample of 15- to 44-year-olds who had ever used or tried to obtain contraception (unweighted n = 2,771)

Variable	Experienced a challenge or delay		
	Yes weighted %	No weighted %	p-value
Current contraceptive method*			< 0.001
No method	16.9	25.1	
Coital-based FABM or withdrawal	13.3	12.9	
Coital-based condoms, spermicide, sponge, diaphragm, cervical cap, Phexxi, EC	13.9	13.4	
SARC (pill, patch, ring, shot)	47.4	23.1	
LARC (IUD, implant)	7.0	20.3	
Vasectomy	1.6	5.3	
Contraceptive user status			< 0.001
Current user			
Using preferred method (content current user)	34.1	51.2	
Wants to use a different method, no method, or stop ASAP	37.5	14.5	
Uncertain current user	11.6	9.3	
Current non-user			
Content non-user (does not want to use a method)	4.9	16.1	
Prospective user (wants to be using a method)	8.5	4.5	
Uncertain non-user	3.5	4.4	
Number of types of discrimination ever experienced in a family planning setting			< 0.001
0 types	28.0	51.5	
1–4 types	35.1	26.2	
5–9 types	36.9	22.3	
Received PCCC from most recent healthcare provider seen for birth control			0.002
Yes	24.4	37.5	
No	75.6	62.5	

*EC* emergency contraception, *FABM* fertility awareness-based methods, *SARC* short-acting reversible contraception, *LARC* long-acting reversible contraception, *PCCC* Person-Centered Contraceptive Counseling, *ASAP* as soon a possible

^*^Some respondents were currently using more than one contraceptive method. Most effective method reported. Abstinence not considered a contraceptive method

Finally, we used logistic regression analyses to investigate the association between previously experiencing a challenge/delay and measures of contraceptive self-efficacy (Table 6). In adjusted analyses, experiencing a challenge/delay in the past year was associated with 86% lower odds that an individual felt very/somewhat confident about obtaining desired contraception compared to those that did not experience a challenge/delay (aOR 0.14, 95% CI 0.07–0.25; Model 1). Experiencing a recent challenge/delay was also associated with our second measure of self-efficacy: perceived ease of switching contraceptive methods (Model 2). In adjusted analyses, experiencing a challenge/delay in the last year was associated with 52% lower odds that an individual felt it would be very/somewhat easy to switch contraceptive methods (aOR 0.48, 95% CI 0.33–0.71).

**Table 6:** Associations between experiencing a challenge or delay and measures of contraceptive self-efficacy among a 2022 national sample of 15- to 44-year-olds who had ever used or tried to obtain contraception (n = 2,771)

	Unadjusted				Adjusted			
	OR	95% CI		p-value	aOR	95% CI		p-value
Model 1: very/somewhat confident about obtaining desired contraception when wanted								
Did not experience a challenge or delay	(Ref.)				(Ref.)			
Did experience a challenge or delay	0.14	0.08	0.24	< 0.001	0.14	0.07	0.25	< 0.001
Model 2: very/somewhat easy to switch contraceptive methods when wanted								
Did not experience a challenge or delay	(Ref.)				(Ref.)			
Did experience a challenge or delay	0.48	0.33	0.71	< 0.001	0.48	0.33	0.71	< 0.001

Logistic regression models adjust for age, race/ethnicity, sexual orientation, educational attainment, insurance, employment status, urbanicity, and largest affordable emergency expense, and accounting for survey weighting

## Discussion

Person-centered contraceptive access entails the opportunity to choose and access a preferred contraceptive method, including starting, switching, and discontinuing when desired [3, 24, 30]. Previous research has found the ability to select and obtain a preferred contraceptive method is associated with consistent and effective contraceptive use and leads to positive health, social, and economic outcomes [64–66]. Our research suggests that experiencing a challenge/delay has implications not only for recent contraceptive access but also influences self-efficacy, potentially inhibiting future ability to obtain and use desired contraception. This can undermine individual reproductive self-determination, as well as SRH equity at the population level. While previous research has focused on either contraceptive barriers or self-efficacy, this is the first study to our knowledge that quantitatively analyzes the relationship between a novel, person-centered metric of experiencing a challenge or delay obtaining desired contraception in the past 12 months and measures of self-efficacy. We use a nationally representative data set, which provides a high level of generalizability to the U.S. population.

In our analyses, the most common types of challenges and delays were logistical and financial, consistent with previous literature [10, 15, 16]. Among those who experienced a challenge/delay obtaining desired contraception, higher proportions were younger, identified as non-heterosexual, had lower educational attainment, and faced greater financial constraints. These findings align with evidence linking structural inequities to contraceptive access barriers [12, 14, 20, 24]. Adjusted analyses reveal that experiencing a challenge or delay was associated with significantly lower odds of contraceptive self-efficacy, particularly confidence in obtaining a desired method and perceived ease of switching methods. To further explore this association, future research should examine additional dimensions of self-efficacy and explore how specific barriers, such as informational versus logistical challenges, influence outcomes. Additionally, future work could consider these associations in contexts with fewer structural barriers, such as countries with universal healthcare systems.

### Implications for policy and practice

To improve person-centered contraceptive access, barriers at the structural level must be addressed, especially regarding logistics and cost, the most frequent challenges experienced in our study. Various strategies can be used to mitigate these challenges, such as advancing community-based approaches to contraceptive access (including over-the-counter, telehealth, and pharmacist-prescribing [24, 27]) and ensuring insurance coverage for these services. Increasing length of supply and expanding scope of practice laws for those who can offer contraception can also be helpful in improving access [67–69].

Additionally, it is important to consider how provider behavior and the quality of contraceptive care can reduce contraceptive barriers and improve self-efficacy. For example, previous research suggests that provider bias and discrimination can inhibit access by influencing patient decision-making and reducing self-efficacy [11, 70, 71]. Therefore, it is crucial that patient needs and preferences are centered in patient-provider discussions regarding contraception, whether for initial contraceptive method selection and preferences, ongoing satisfaction, or desires around switching/discontinuing [67, 72, 73]. This is especially important for young, low-income, and socially marginalized populations, which our research suggests may be more likely to experience barriers to contraceptive access. Institutions and individuals must remain conscious of how their own beliefs and practices can show up as assumptions about what is “best” when it comes to family size, pregnancy spacing, and approaches to fertility and contraception [74].

### Limitations

There were several limitations to this study. First, although we asked about the type of challenge/delay experienced, our survey did not include follow-up questions to garner additional details. Therefore, we are unable to ascertain when, where, with whom, or how long ago the respondent experienced challenges or delays—important details that future research should address to shed light on strategies to address contraceptive access barriers. Additionally, given that the survey question only asked about challenges/delays experienced in the past 12 months, we do not have information about barriers experienced outside that time frame, which could also impact self-efficacy. Contraceptive self-efficacy is a complex construct. While we included straightforward questions in our survey related to the key aspects of self-efficacy, our measures likely do not fully capture the nuance of this construct. Last, we excluded from the sample people not assigned female sex at birth and those using permanent contraception or who were otherwise infecund, so our results may not generalize to these populations who may have also experienced contraceptive challenges or delays.

## Conclusions

Person-centered metrics of contraceptive access, compared to traditional measures focused on contraceptive use and services performed, are crucial to achieve SRH equity. This study, utilizing various person-centered metrics, found encountering challenges or delays has implications not only for recent contraceptive access, but also influences self-efficacy—potentially inhibiting future ability to obtain and use desired contraception. Given these short- and long-term impacts, we urge policymakers, clinicians, and researchers to support policies and practices that reduce barriers to person-centered contraceptive access.

## Data Availability

The data and codebook will be publicly available from the Open Science Framework at https://osf.io/wvb5m/

## Abbreviations

SRH: Sexual and reproductive health
BIPOC: Black, indigenous, and people of color
IUD: Intrauterine device
SARC: Short-acting reversible contraception
LARC: Long-acting reversible contraception
FABM: Fertility awareness-based methods
PCCC: Person-centered contraceptive counseling
aOR: Adjusted odds ratio
CI: Confidence interval
ASAP: As soon as possible
